# Supplementary material for: AMPK activation counteracts cardiac hypertrophy by reducing O-GlcNAcylation
Source: Nat Commun. 2018 Jan 25;9:374. doi: 10.1038/s41467-017-02795-4 (PMC5785516; doi:10.1038/s41467-017-02795-4)
Supplement: Supplementary file 1 — Supplementary Information [file 41467_2017_2795_MOESM1_ESM.pdf]

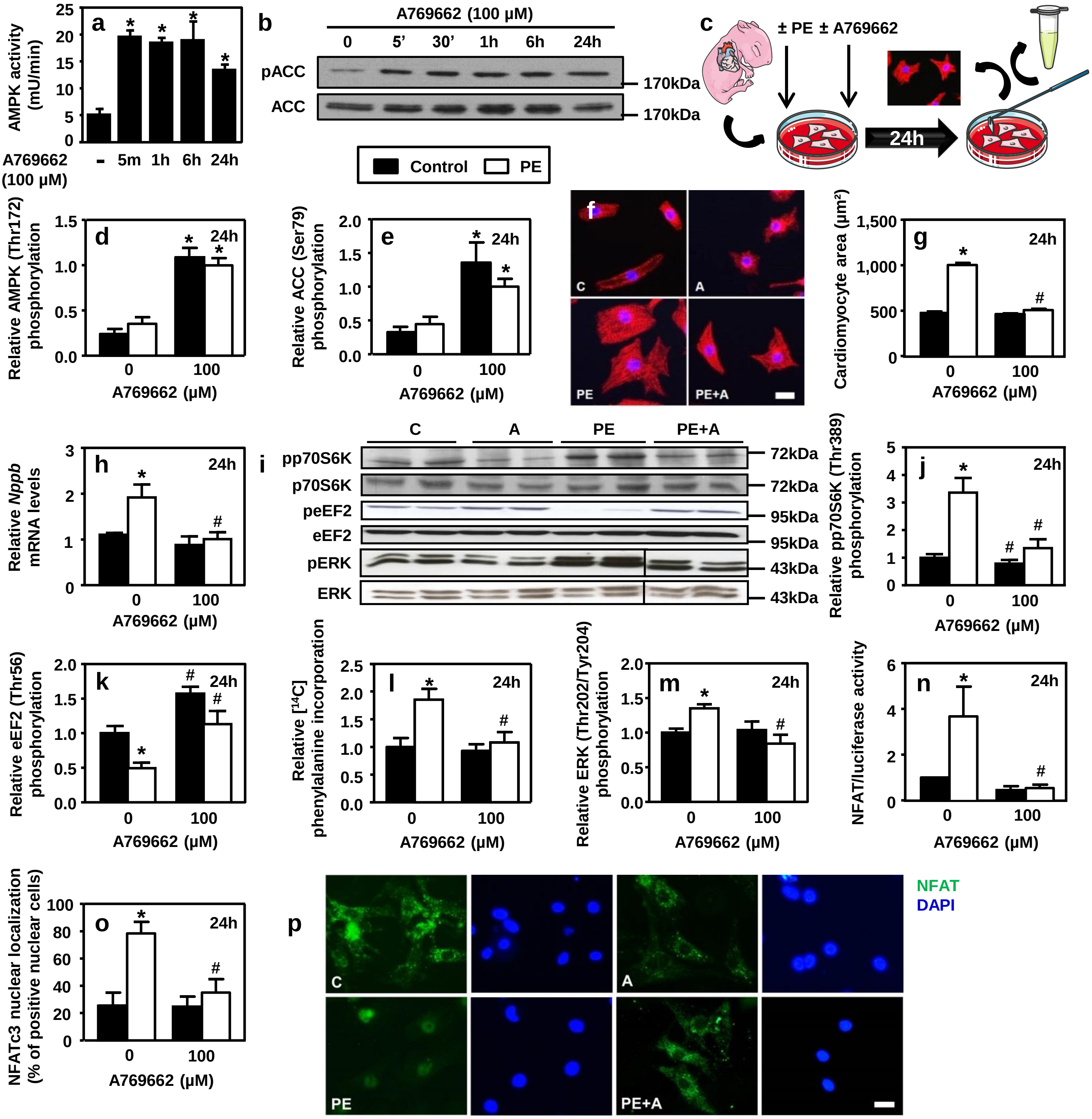

Supplementary Figure 1: High concentration of A769662 inhibits phenylephrine-induced NRVM hypertrophy and concomitantly modulates the previously identified AMPK downstream targets. (**a**) Time course of AMPK activation by A769662 (100 µM). N=5. (**b**) Representative immunoblot of ACC^{Ser79} phosphorylation from time course curve of A769662 (100 µM). (**c**) Schematic protocol followed in (**d**-**p**). (**d**-**p**) NRVMs were treated with (open bars) or without (solid bars) phenylephrine (PE, 20 µM) in the presence or absence of A769662 (A, 100 µM) for 24 h except for ERK1/2 phosphorylation which has been evaluated after 1h. (**d**) Quantification of AMPKα^{Thr172} phosphorylation. N=5. (**e**) Quantification of ACC^{Ser79} phosphorylation. N=5. (**f**,**g**) Representative images and quantification of cardiomyocyte area evaluated after α-actinin immunostaining. Scale bar, 20 µm. N=5. (**h**) Relative *Nppb* expression. N=4. (**i**) Representative immunoblots of p70S6K^{Thr389}, eEF2^{Thr56} and ERK$^{Thr202/Tyr204}$ phosphorylation and their respective total content. (**j**) Quantification of p70S6K^{Thr389} phosphorylation. N=11. (**k**) Quantification of eEF2^{Thr56} phosphorylation. N=7. (**l**) Amino acids incorporation into proteins measured by [^{14}C]-phenylalanine incorporation. N=5. (**m**) Quantification of ERK$^{Thr202/Tyr204}$ phosphorylation. N=10. (**n**) Evaluation of NFAT transcriptional activity by luciferase activity. N=5. (**o**,**p**) Representative images and quantitative representation of NFATc3 translocation to the nucleus. Scale bar, 20 µm. N=5. Data in **a**-**o** are mean ± s.e.m. The data were analyzed using One-way ANOVA followed by Bonferroni post-test in **a** and Two-way ANOVA followed by Bonferroni post-test in **d**-**e**, **g**-**h** and **j**-**o**. *p<0.05 vs. untreated cells, #p<0.05 vs. corresponding PE-treated cells.

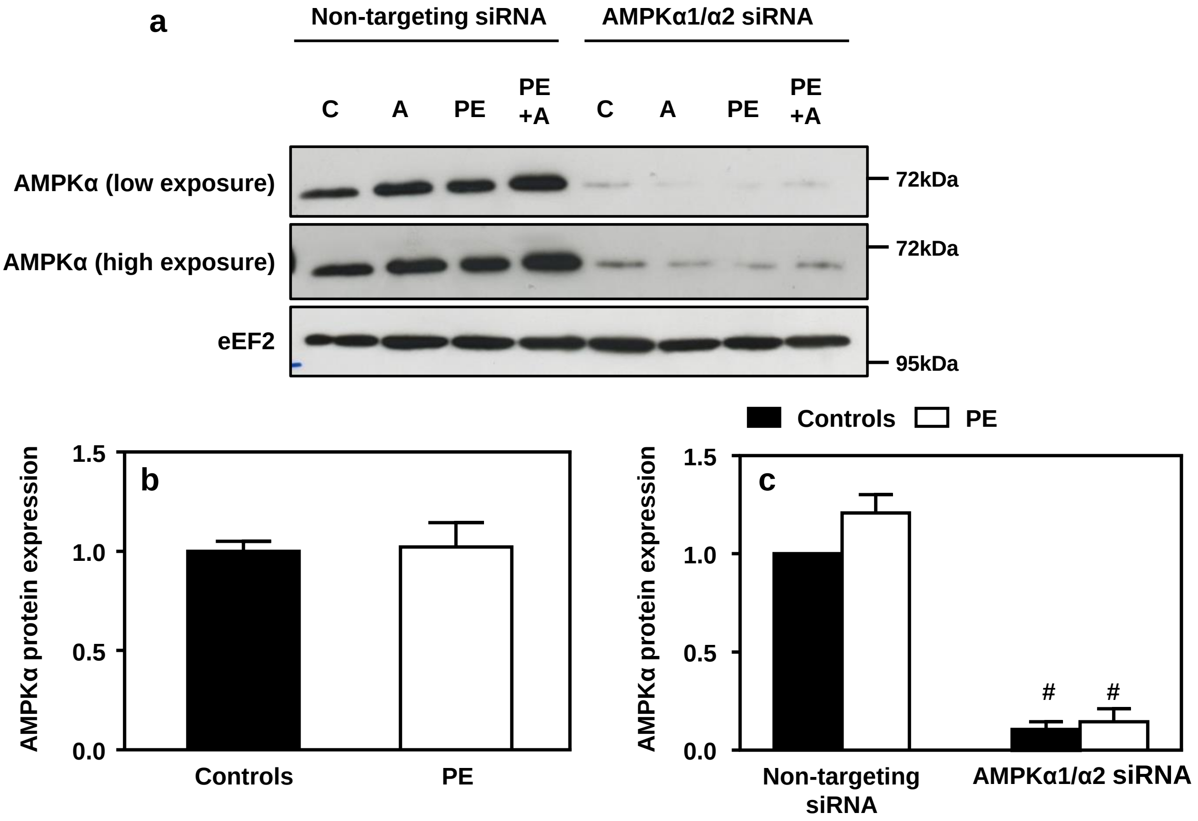

Supplementary Figure 2: Phenylephrine treatment does not modify AMPKα expression. Neonatal cardiomyocytes were transfected with non-targeting siRNA and with siRNA directed against both AMPKα subunits for 66h and then treated with A769662 (12.5 µM) and phenylephrine (PE, 20 µM) for 24h. (**a**) Representative immunoblots of AMPKα protein expression at low and high exposure. (**b**) Quantification of AMPKα protein expression in basal conditions. N=8. (**c**) Quantification of AMPKα protein expression in non-targeting siRNA conditions and in AMPKα1/α2 siRNA conditions. N=4. Data in **b**-**c** are mean ± s.e.m. The data were analyzed using Two-way ANOVA followed by Bonferroni post-test in **c** and using unpaired Student's t-test in **b**. #p<0.05 vs. non-targeting control and PE conditions. C = control, A = A769662, PE = phenylephrine and PE+A = phenylephrine + A769662, eEF2 was used as loading control.

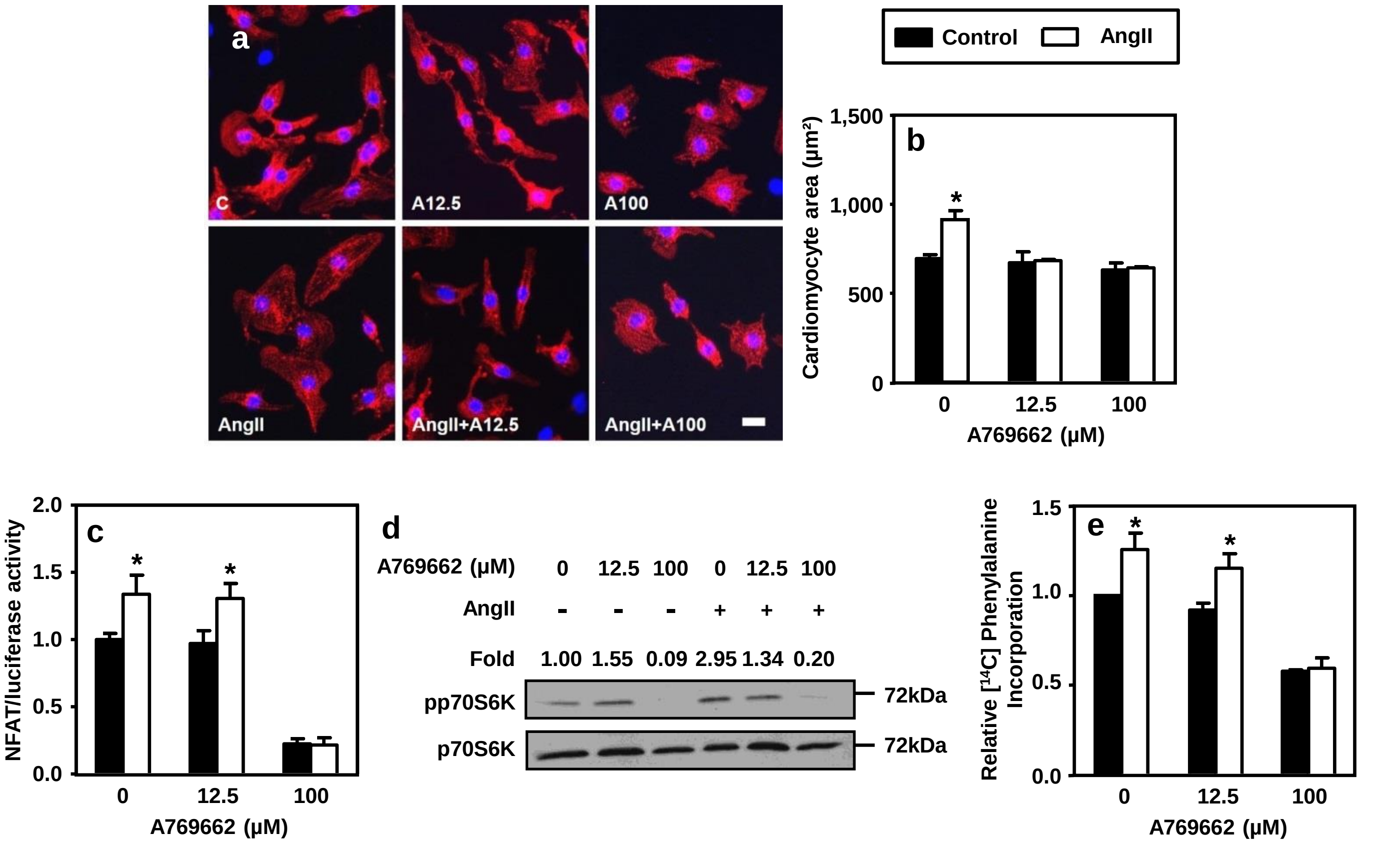

Supplementary Figure 3: Low concentration of A769662 inhibits Angiotensin II-induced hypertrophy in NRVMs without acting on the previously identified AMPK downstream targets. (**a**-**e**) NRVMs were treated with (open bars) or without (solid bars) Angiotensin II (AngII, 100 nM) in the presence or absence of A769662 (0, 12.5 or 100 µM) for 24 h. (**a**-**b**) Representative images and quantification of cardiomyocyte area evaluated after α-actinin immunostaining. Scale bar, 20 µm. N=3. (**c**) Evaluation of NFAT transcriptional activity by luciferase activity. N=3. (**d**) Representative immunoblot of p70S6K^{Thr389} phosphorylation. The fold increase relative to control condition is presented above the gel. (**e**) Amino acids incorporation into proteins measured by [^{14}C]-phenylalanine incorporation. N=5. Data in **b**, **c**, **e** are expressed as mean ± s.e.m. and were analyzed using Two-way ANOVA followed by Bonferroni post-test. *p<0.05 vs. untreated cells.

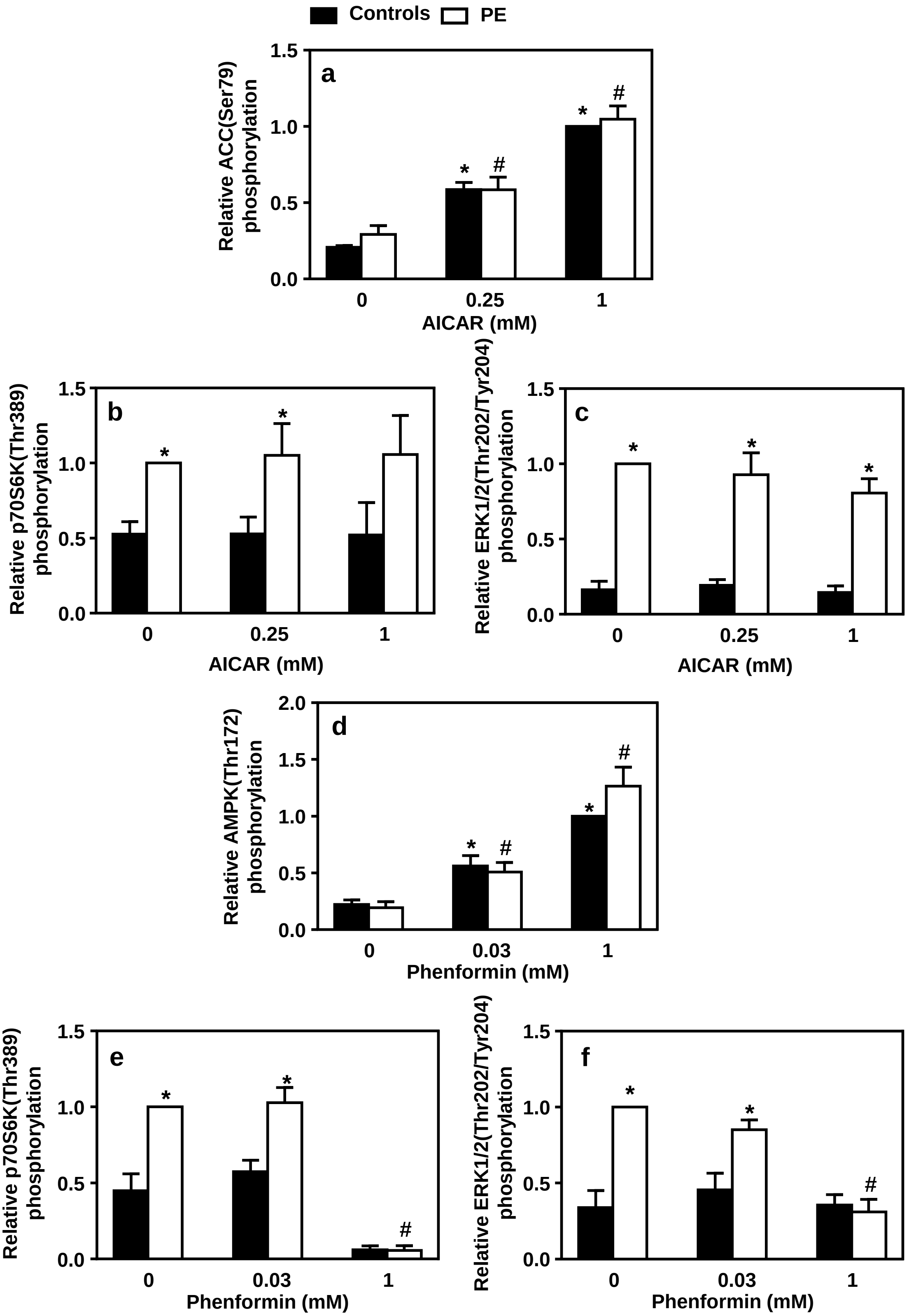

Supplementary Figure 4: Low concentration of AICAr and phenformin prevents phenylephrine-induced NRVM hypertrophy without acting on the previously identified AMPK downstream targets. (**a**-**b**) NRVMs were treated with (open bars) or without (solid bars) phenylephrine (PE, 20 µM) in the presence or not of AICAr (from 0.25 to 1 mM) for 24h. (**a**) Quantification of ACC^{Ser79} phosphorylation. N=5. (**b**) Quantification of $p70S6K^{Thr389}$ phosphorylation. N=4-8. (**c**) NRVMs were treated with (open bars) or without (solid bars) phenylephrine (PE, 20 µM) in the presence or not of AICAr (from 0.25 to 1 mM) for 1h. Quantification of $ERK^{Thr202/Tyr204}$ phosphorylation. N=4. (**d**-**e**) NRVMs were treated with (open bars) or without (solid bars) phenylephrine (PE, 20 µM) in the presence or not of phenformin (from 0.03 to 1 mM) for 24h. (**d**) Quantification of $AMPK^{Thr172}$ phosphorylation. N=4. (**e**) Quantification of $p70S6K^{Thr389}$ phosphorylation. N=3-4. (**f**) NRVMs were treated with (open bars) or without (solid bars) phenylephrine (PE, 20 µM) in the presence or not of phenformin (from 0.03 to 1 mM) for 1h. Quantification of $ERK^{Thr202/Tyr204}$ phosphorylation. N=4. Data in **a**-**f** are expressed as mean ± s.e.m. and were analyzed using Two-way ANOVA followed by Bonferroni post-test. *$p<0.05$ vs. untreated cells, #$p<0.05$ vs. PE-treated cells.

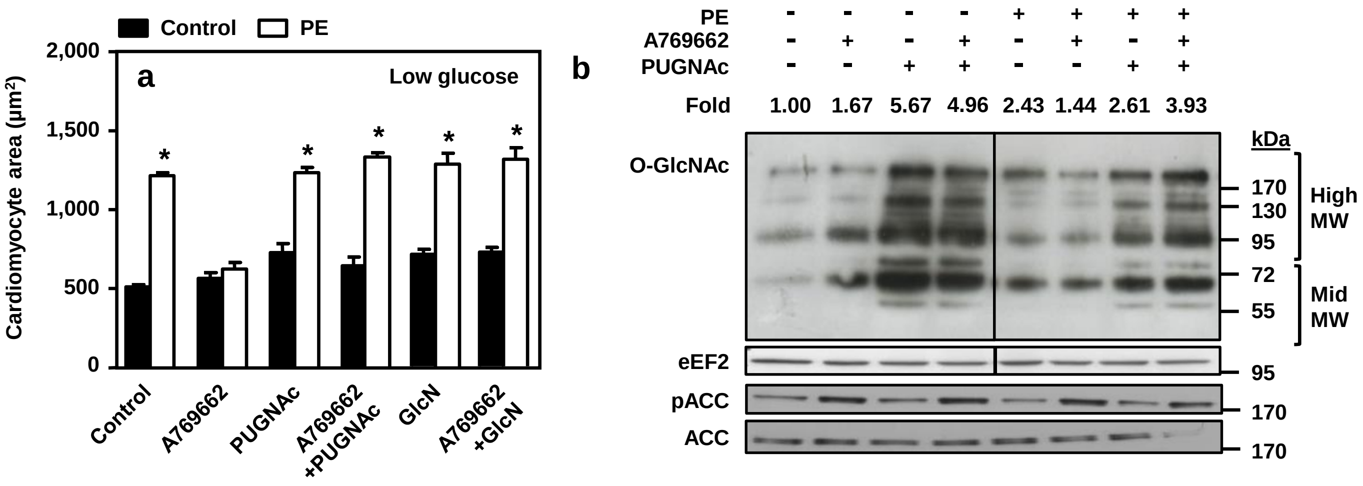

Supplementary Figure 5: Submaximal A769662 concentration inhibits phenylephrine-induced hypertrophy in NRVMs independently of extracellular glucose concentration. (**a**-**b**) NRVMs were treated with (open bars) or without (solid bars) phenylephrine (PE, 20 µM) in the presence or absence of A769662 (12.5 µM), PUGNAc (50 µM) and/or GlcN (5 mM) for 24 h under low glucose (5 mM) condition. (**a**) Quantification of cardiomyocyte area evaluated after α-actinin immunostaining. N=3. (**b**) Representative immunoblot of protein O-GlcNAcylation levels. The fold increase relative to control condition is presented above the gel. Data in **a** are expressed as mean ± s.e.m. and were analyzed using Two-way ANOVA followed by Bonferroni post-test. *p<0.05 vs. untreated cells. eEF2 was used as loading control.

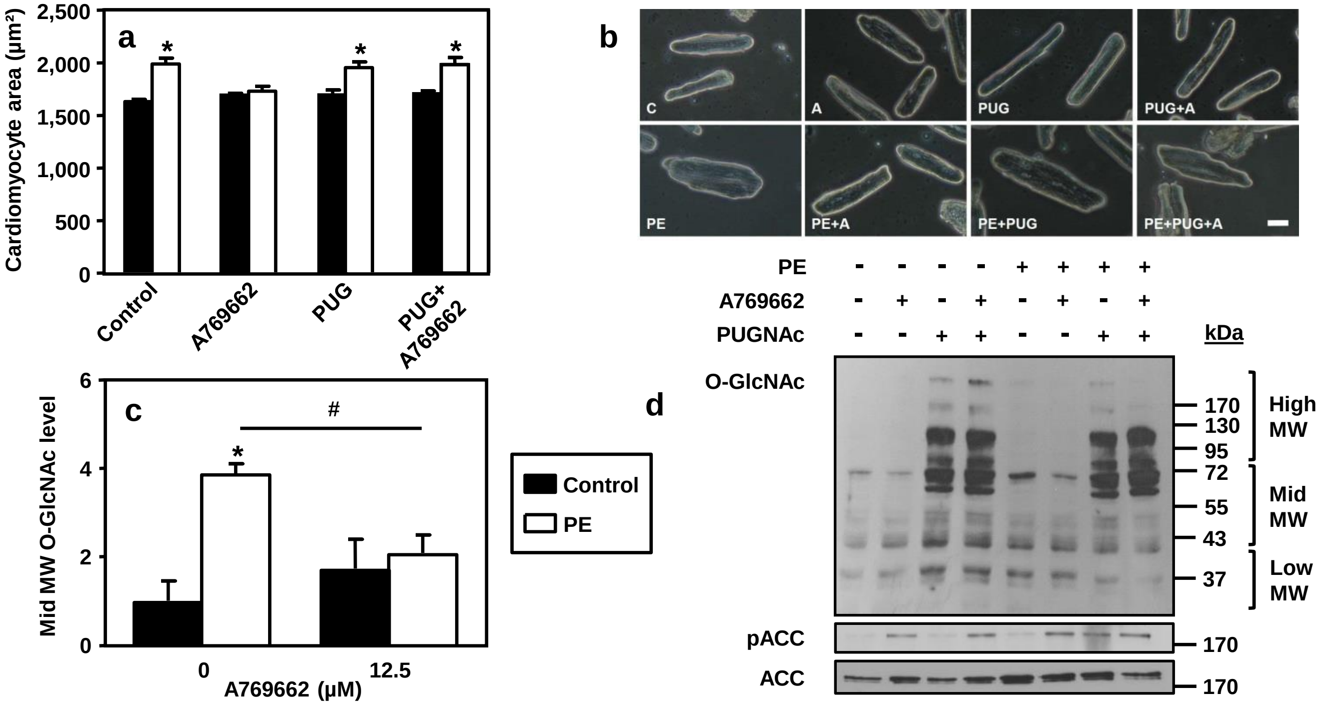

Supplementary Figure 6: Submaximal A769662 concentration inhibits phenylephrine-induced hypertrophy in ARVMs by reducing O-GlcNAcylation levels. (**a**-**d**) ARVMs were treated with (open bars) or without (solid bars) phenylephrine (PE, 100 µM) in the presence or not of A769662 (12.5 µM) and PUGNAc (25 µM) for 48h. (**a**-**b**) Representative images and quantification of cardiomyocyte area under contrast phase microscope. N=4. (**c**) Quantification of protein O-GlcNAcylation levels. N=3. (**d**) Representative immunoblot of protein O-GlcNAcylation levels and ACC^{Ser79} phosphorylation. Data in **a**, **c** are expressed as mean ± s.e.m. and were analyzed using Two-way ANOVA followed by Bonferroni post-test. *p<0.05 vs. untreated cells, #p<0.05 vs PE-treated cells.

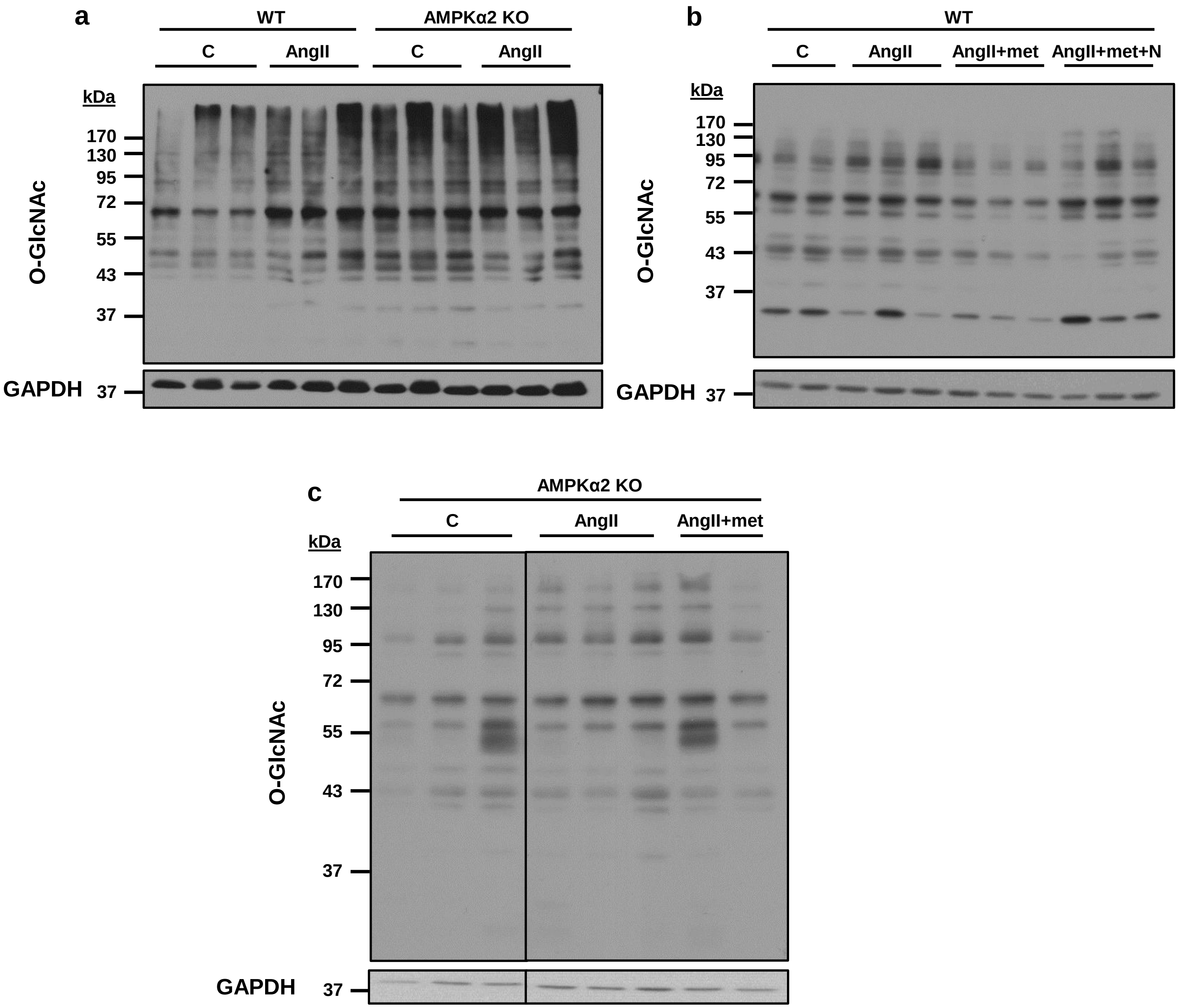

Supplementary Figure 7: Representative immunoblots of O-GlcNAcylation levels in mouse heart samples pre-cleared with PGS. (**a**-**c**) Whole hearts were first pre-cleared with PGS in order to prevent the interaction of the secondary antibody (produced in mouse) with endogenous immunoglobulins. Total heart lysate (140 µg of protein) were incubated with 5 µl of PGS for 1 hour. 20 µg of pre-cleared proteins were then used for immunoblotting with primary anti-O-GlcNAc antibody and secondary anti-mouse antibody. (**a**) WT and AMPKα2 KO mice were treated with or without angiotensin II (AngII, 2 mg/kg/d) for 5 days. (**b**) WT mice were treated with or without angiotensin II (AngII, 2 mg/kg/d), metformin (met, 200 mg/kg/d) and NButGT (50 mg/kg/d) for 5 days. (**c**) AMPKα2 KO mice were treated with or without angiotensin II (AngII, 2 mg/kg/d) and metformin (met, 200 mg/kg/d) for 5 days. The line indicates that one sample has been removed since the PGS pre-clearing did not work efficiently. GAPDH was used as loading control.

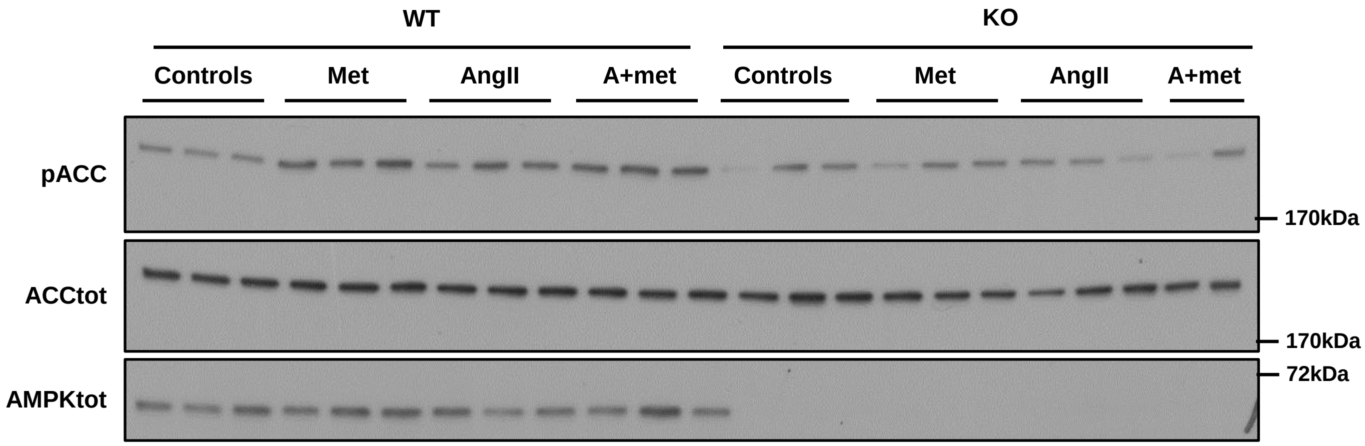

Supplementary Figure 8: Metformin activates AMPK signaling in WT mouse hearts but not in AMPKα2 KO mouse hearts. WT and AMPKα2 KO mice were treated with metformin (200 mg/kg/d) for 5 days. Representative immunoblots of ACC^{Ser79} phosphorylation, ACC protein expression and AMPKα protein expression.

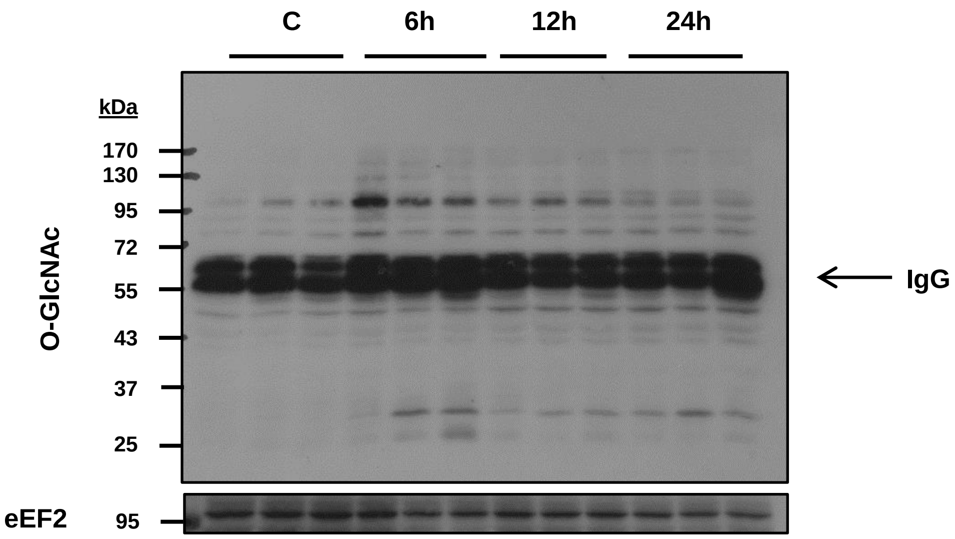

Supplementary Figure 9: Single dose of NButGT induces long-term increase in O-GlcNAc levels. Representative immunoblot of O-GlcNAcylated proteins in a not pre-cleared extract of WT mouse heart treated with NButGT (50 mg/kg) for increasing times. eEF2 was used as loading control.

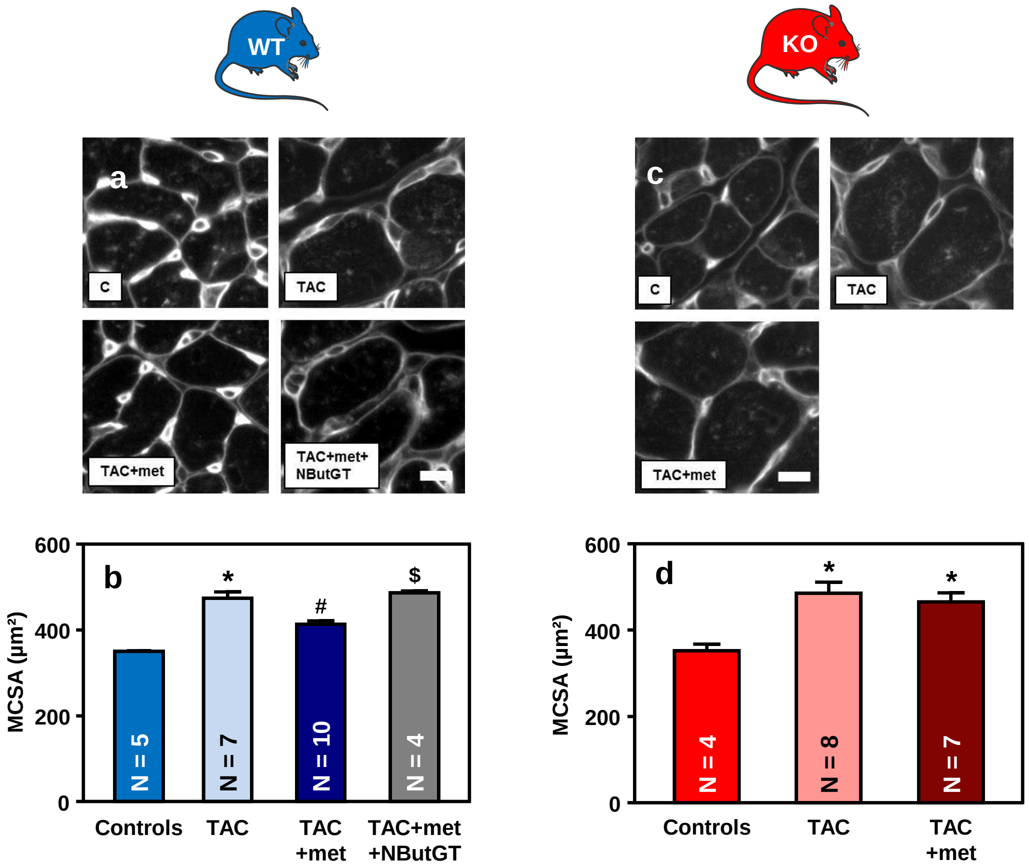

Supplementary Figure 10: AMPK activation by metformin inhibits transaortic constriction-induced cardiac hypertrophy in WT but not in AMPKα2 KO mice and NButGT reverses metformin action in WT mice. (**a**-**b**) After transaortic constriction (TAC) surgery, WT mice were treated with or without metformin (met, 200 mg/kg/d) and NButGT (50 mg/kg/d) for 3 weeks. Representative images and quantification of myocyte cross sectional area evaluated after WGA staining. (**c**-**d**) After TAC surgery, AMPKα2 KO mice were treated with or without metformin (200 mg/kg/d) for 3 weeks. Representative images and quantification of myocyte cross sectional area evaluated after WGA staining. Data in **b**, **d** are expressed as mean ± s.e.m. and were analyzed using One-way ANOVA followed by Bonferroni post-test. *p<0.05 vs. untreated mice, #p<0.05 vs. TAC mice, \$$p$<0.05 vs TAC+met mice.

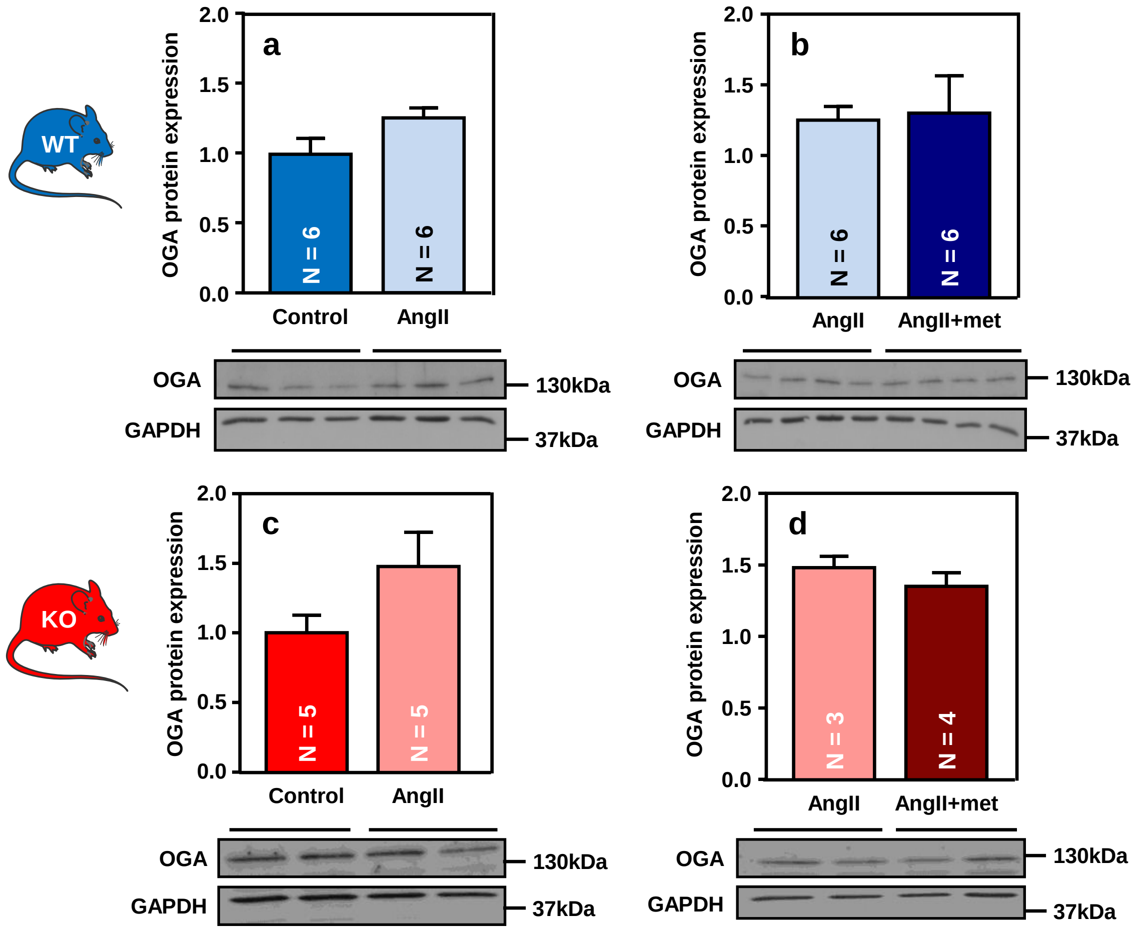

Supplementary Figure 11: Cardiac OGA protein levels are not modified by angiotensin II and metformin treatments. Data are relative to respective controls and are mean ± s.e.m. of n=5-7 hearts from WT (blue) and KO (red) mice treated for 5 days with angiotensin II (AngII, 2 mg/kg/d) and with or without metformin (200 mg/kg/d). GAPDH was used as loading control.

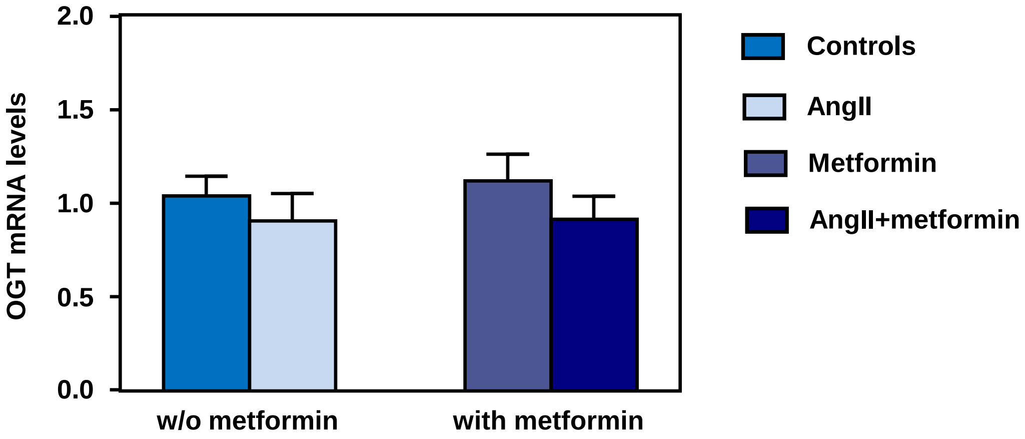

Supplementary Figure 12: Cardiac OGT mRNA levels are not modified by angiotensin II and metformin treatments. Data are relative to controls and are mean ± s.e.m. of n=6-9 hearts from WT mice treated or not with angiotensin II (AngII, 2 mg/kg/d) and metformin (200 mg/kg/d) for 5 days.

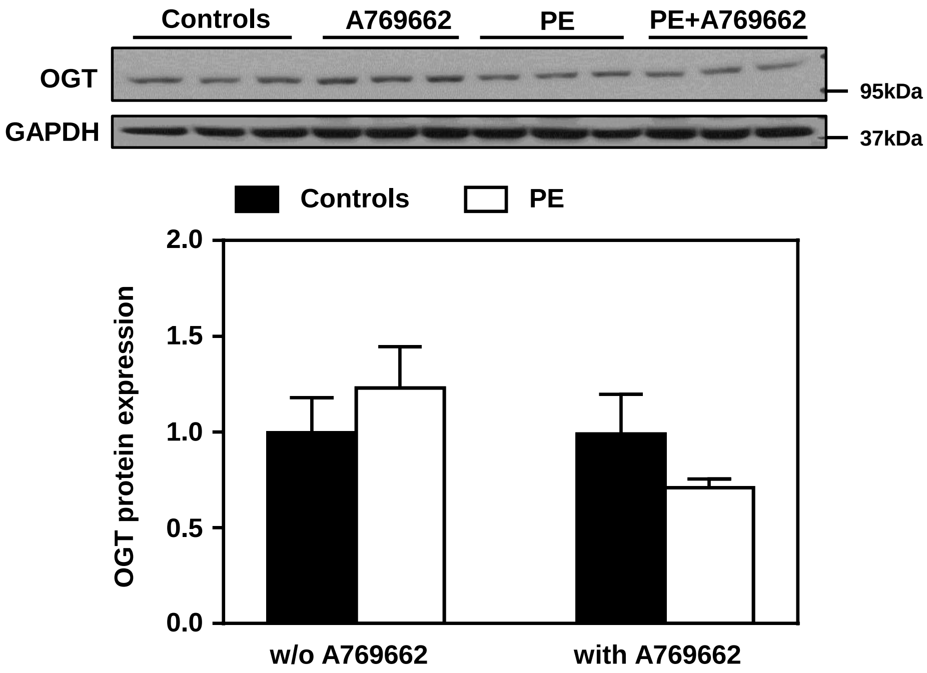

Supplementary Figure 13: OGT protein levels are not altered by phenylephrine and A769662 treatments in NRVMs. Representative immunoblot and quantification of OGT protein levels in NRVMs treated with or without phenylephrine (PE, 20 µM) and A769662 (12.5 µM) for 24h. N=3. Data are expressed as mean ± s.e.m. GAPDH was used as loading control.

Fig. 1A

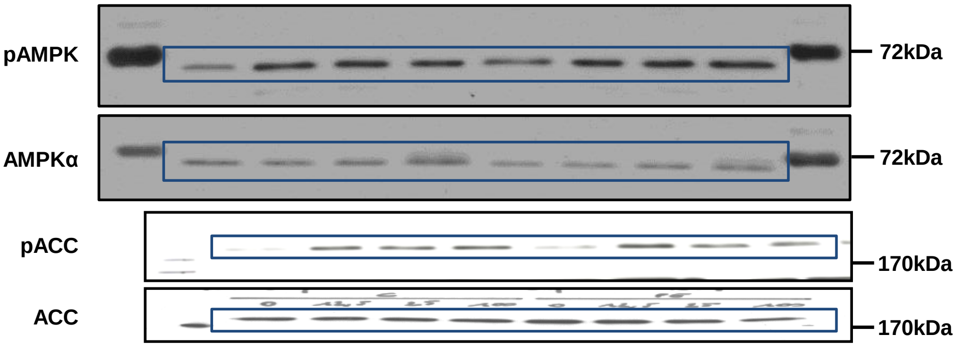

Fig. 1F

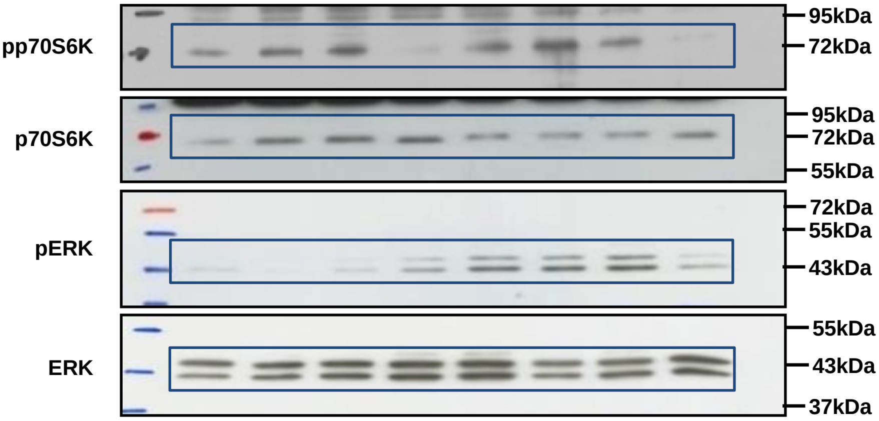

Fig. 1I

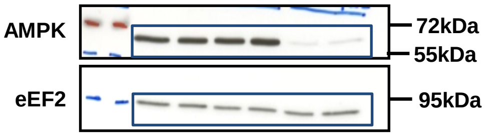

Fig. 1J

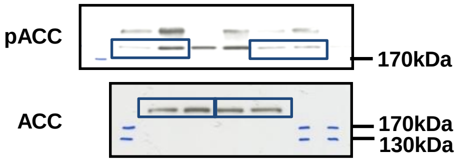

Fig. 2C

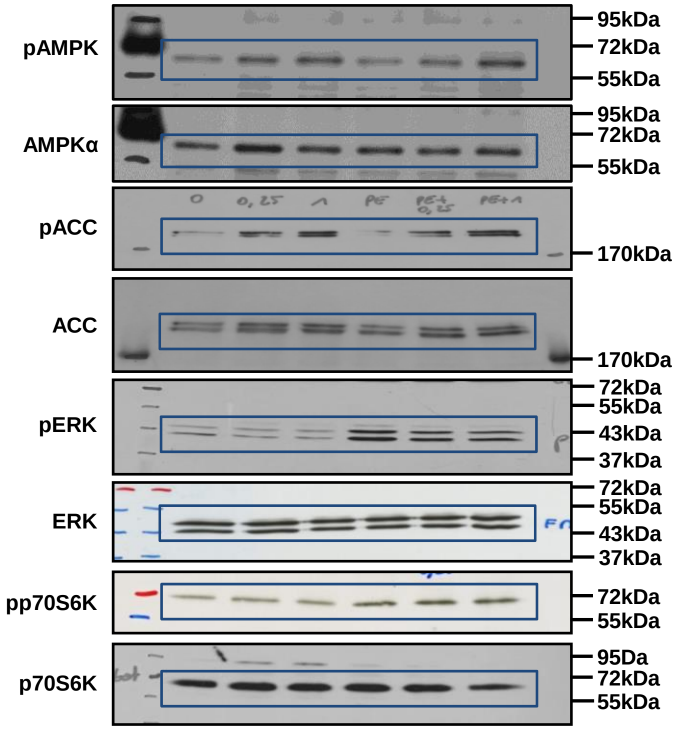

Fig. 2H

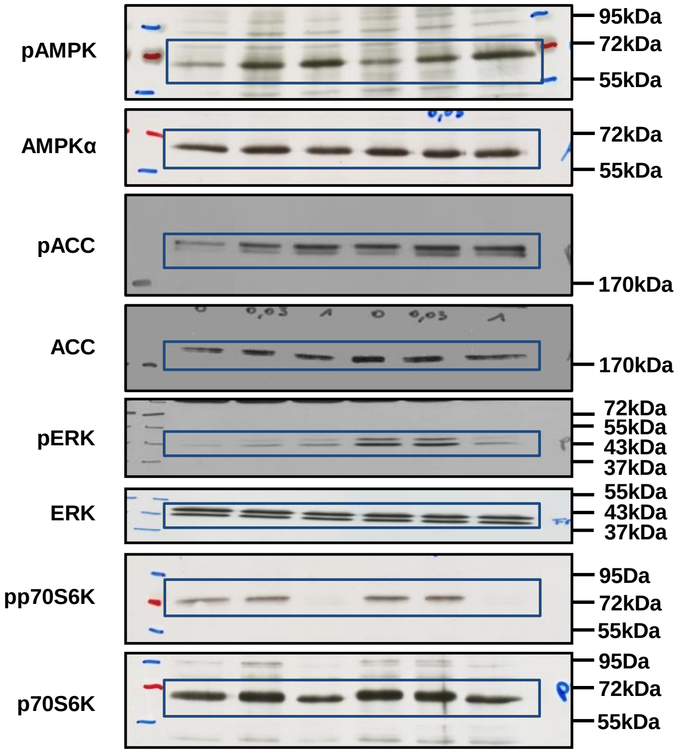

Fig. 3B

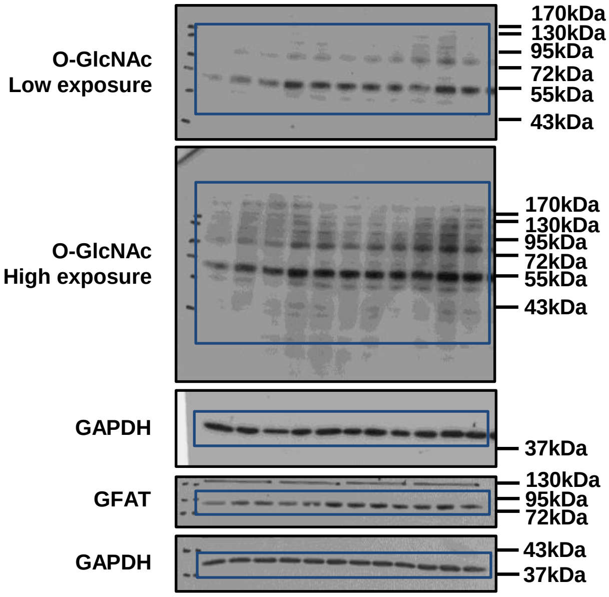

Fig. 3F

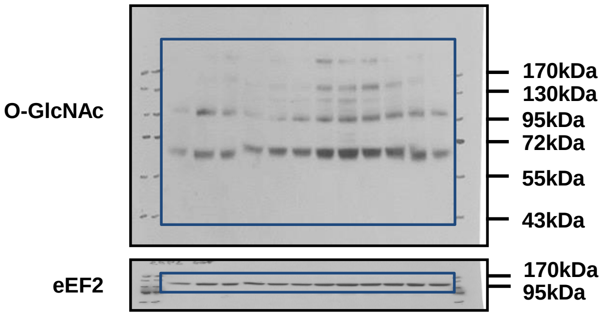

Fig. 3J

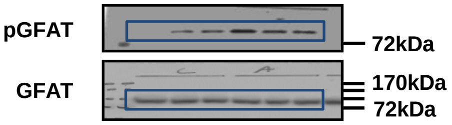

Fig. 4A

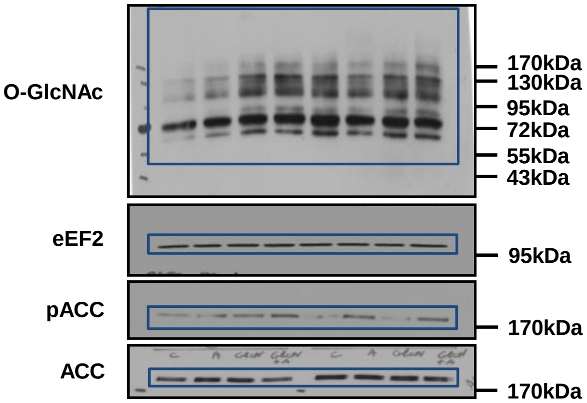

Fig. 4C

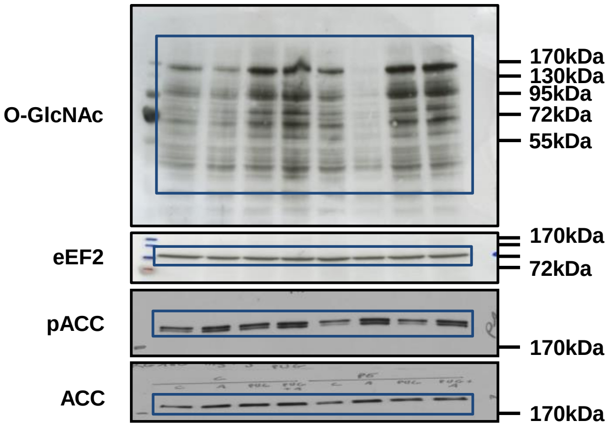

Fig. 5

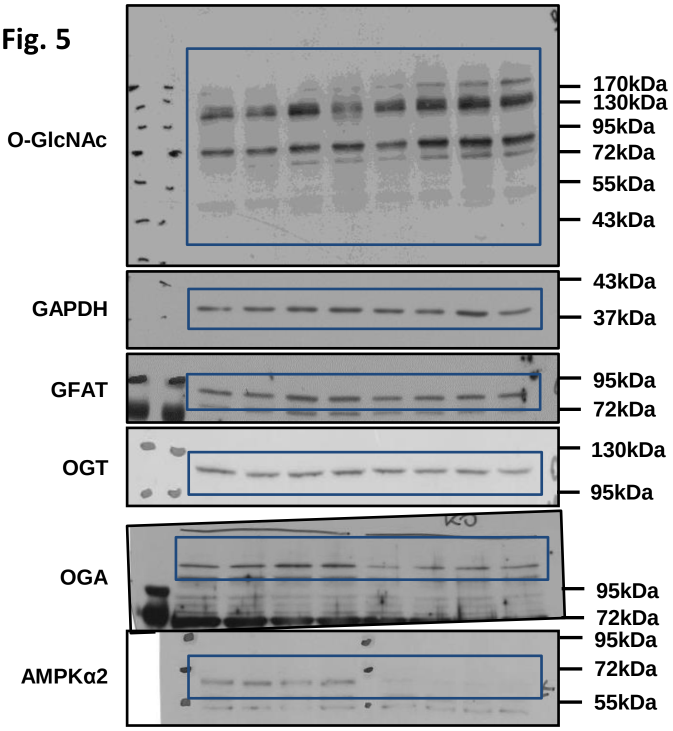

Fig. 6

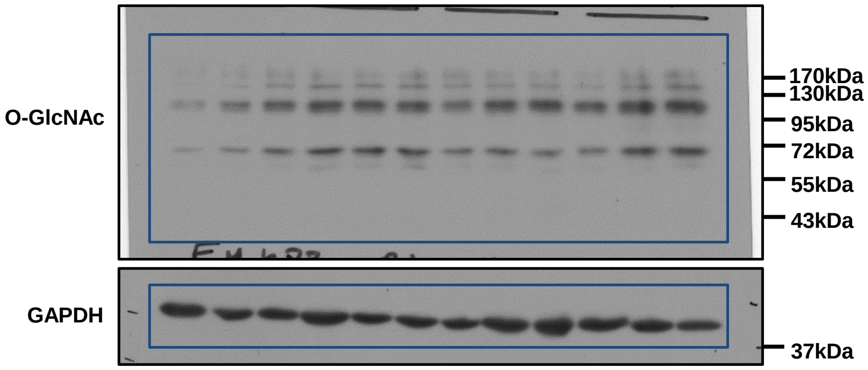

Fig. 8D

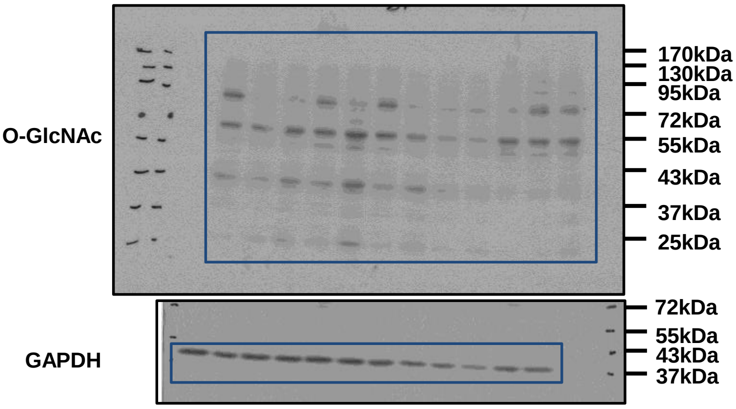

Fig. 8F

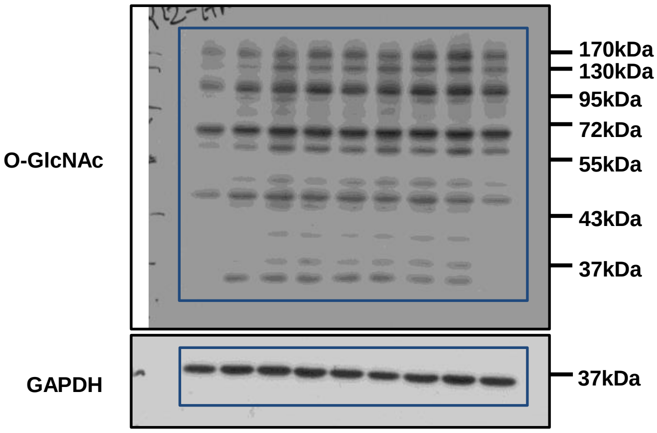

Fig. 9A

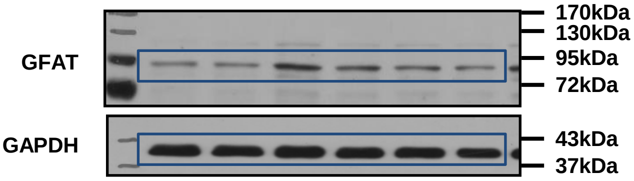

Fig. 9F

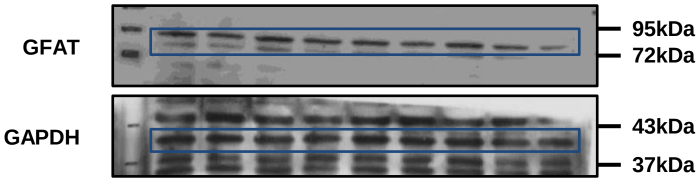

Fig. 9B

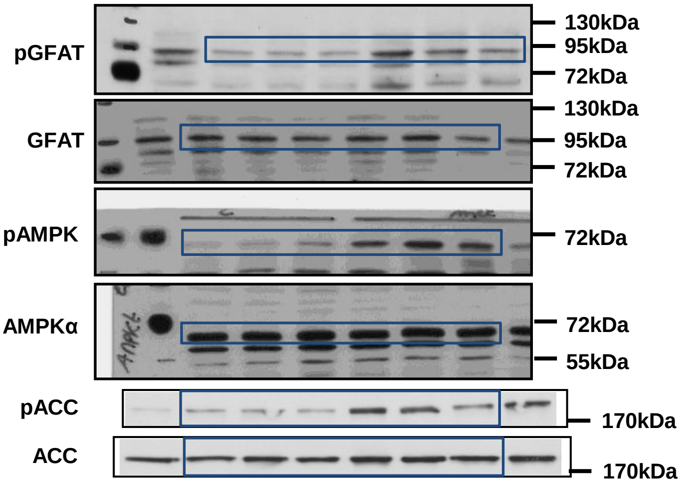

Fig. 9G

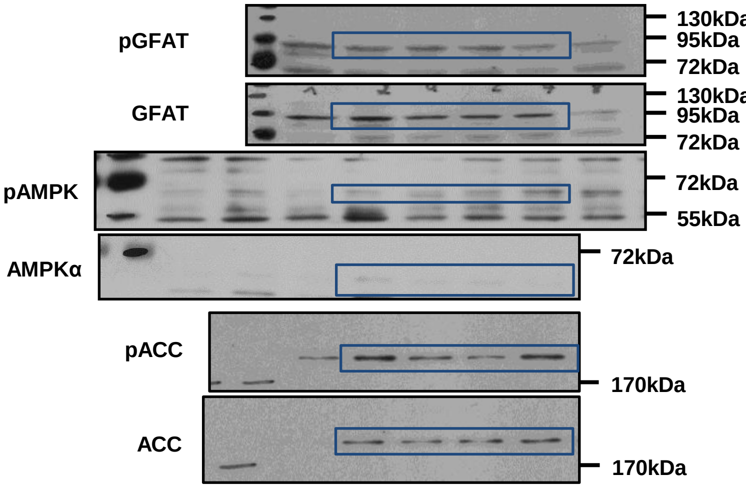

Fig. 10 A and B

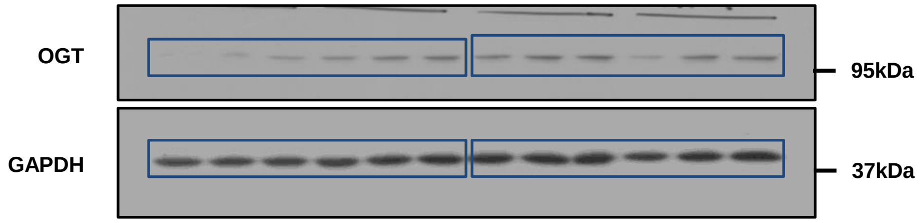

Fig. 10 C

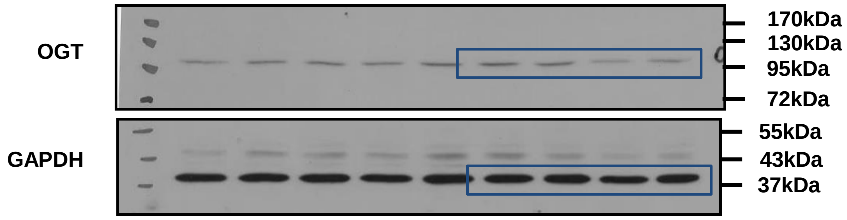

Fig. 10 D

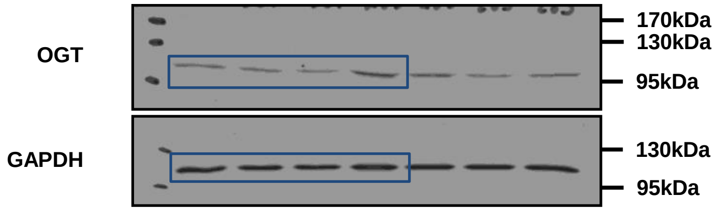

Fig. 10 E

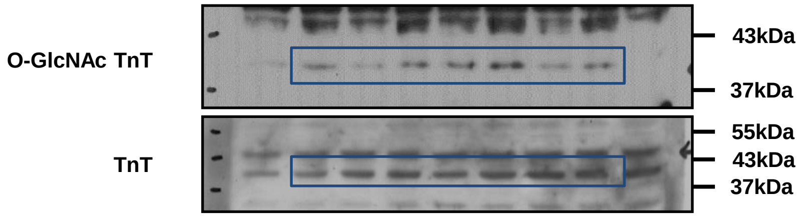

Fig. 10 F

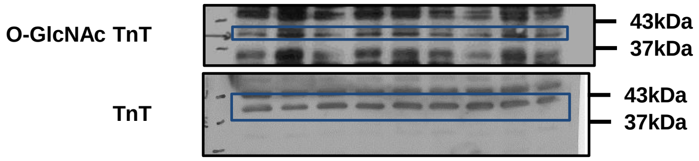

Supplementary Figure 14: Uncropped version of all immunoblots found in main figures.

Supp Fig. 1B

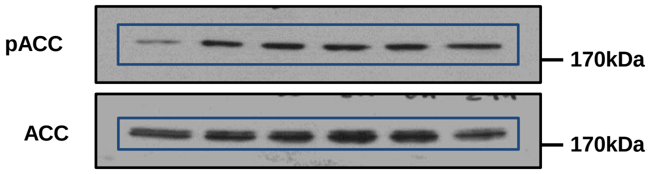

Supp Fig. 1I

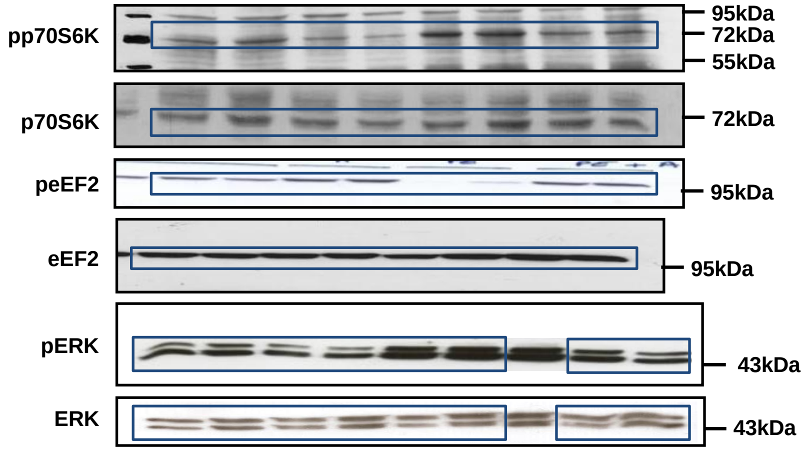

Supp Fig. 2A

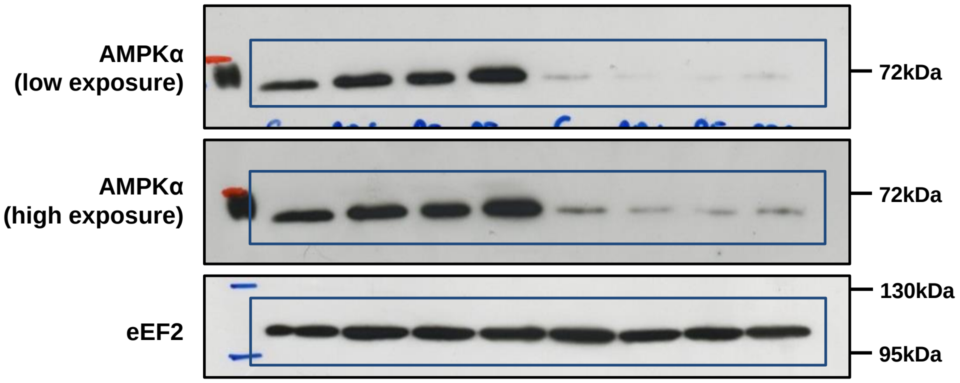

Supp Fig. 3D

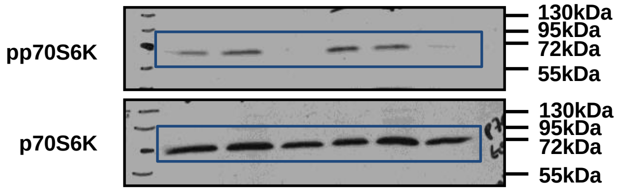

Supp Fig. 5B

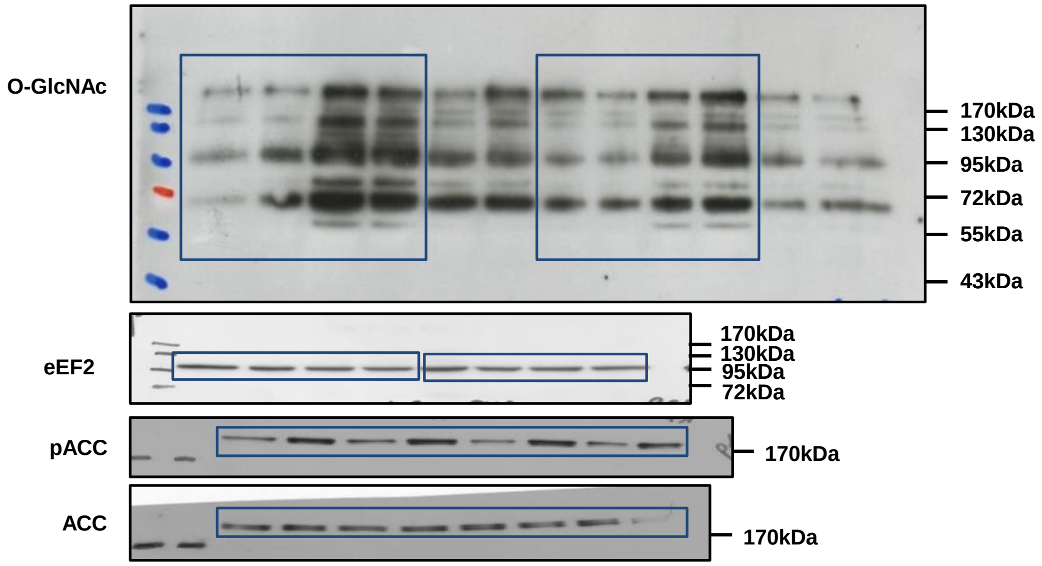

Supp Fig. 6D

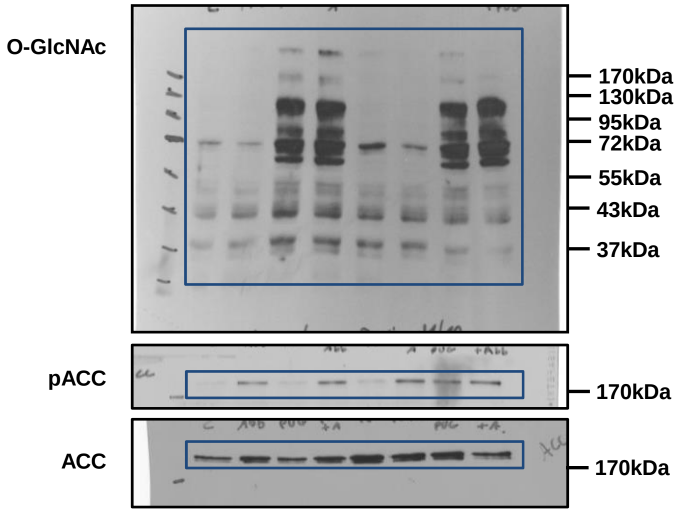

Supp Fig. 7A

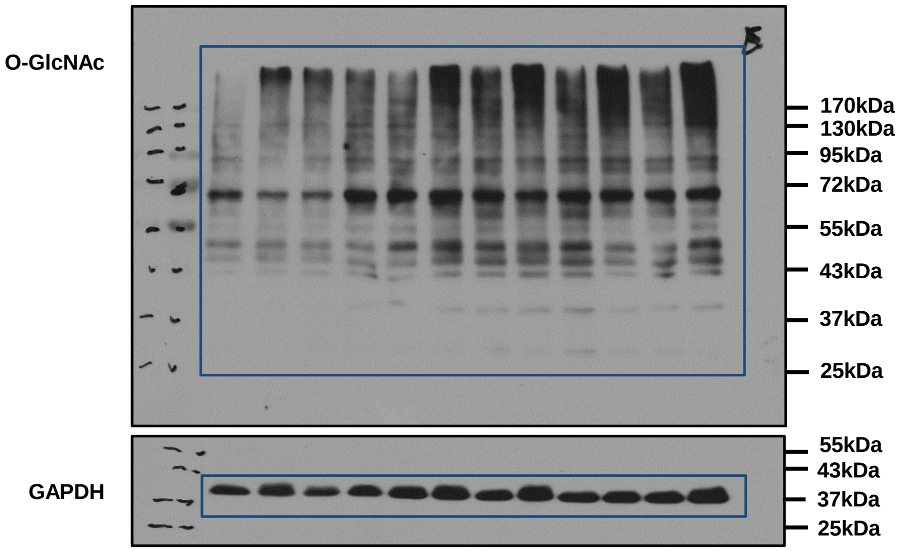

Supp Fig. 7B

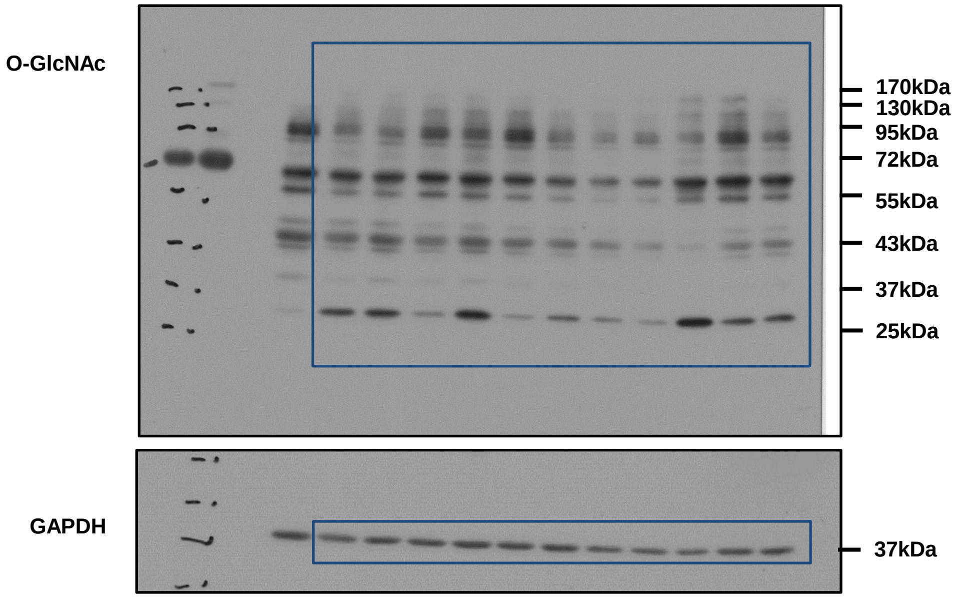

Supp Fig. 7C

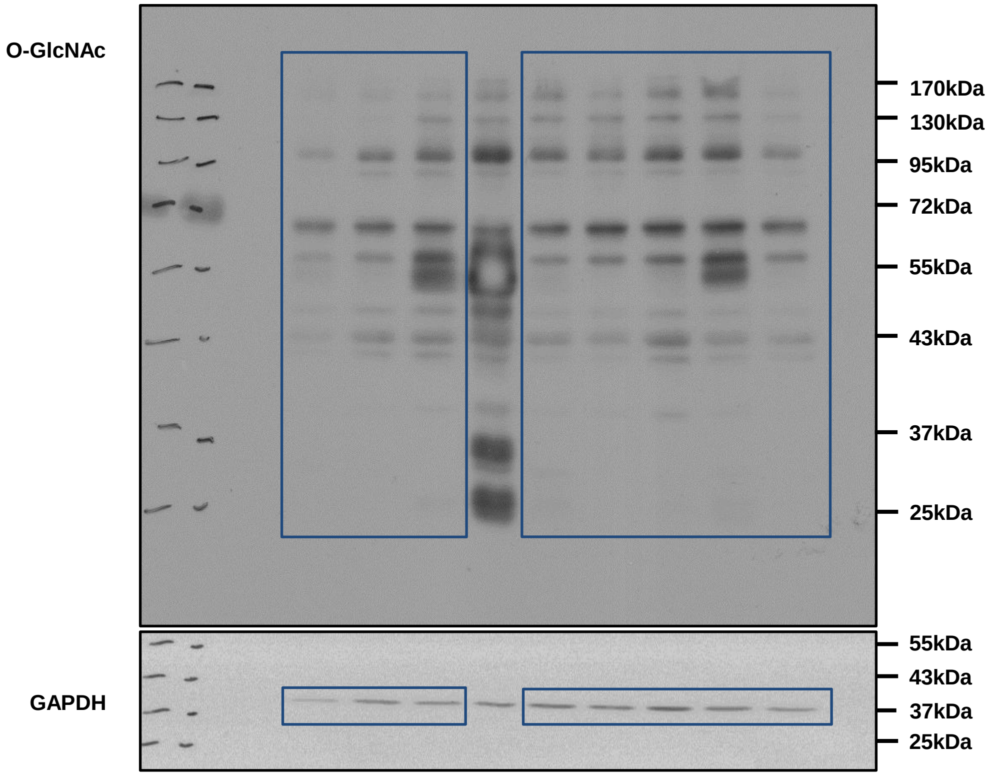

Supp Fig. 8

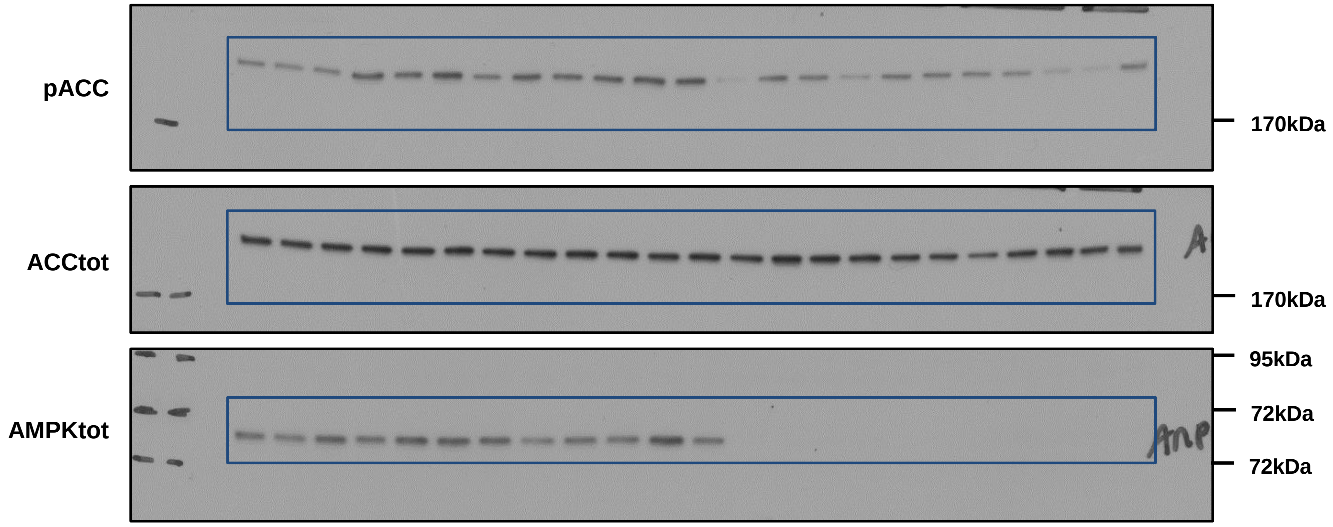

Supp Fig. 9

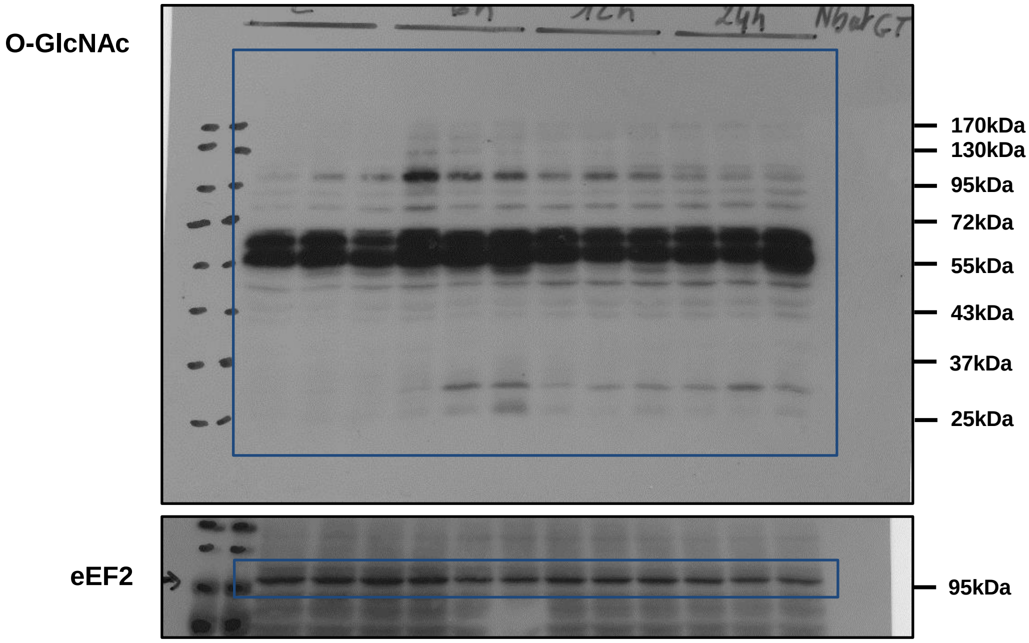

Supp Fig. 11A

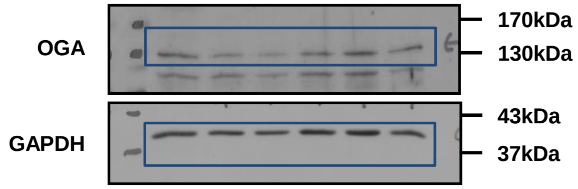

Supp Fig. 11B

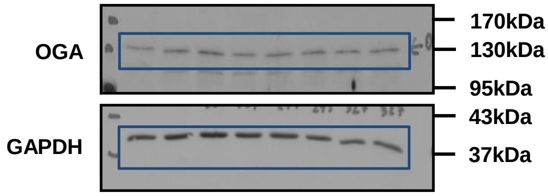

Supp Fig. 11C

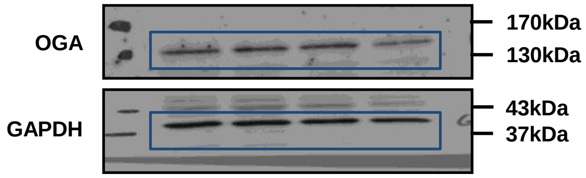

Supp Fig. 11D

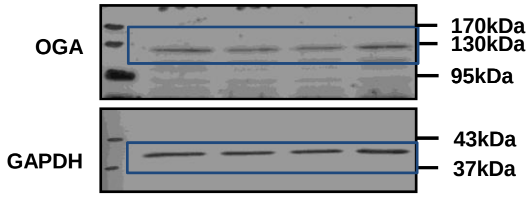

Supp Fig. 13

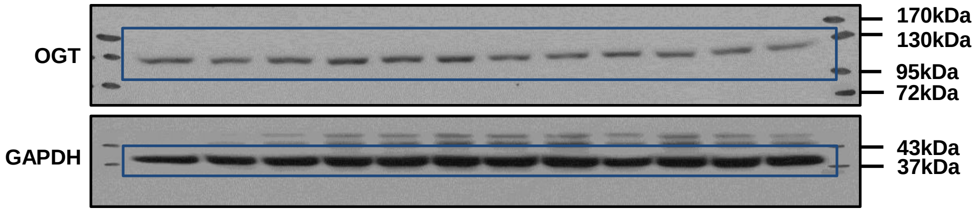

Supplementary Figure 15: Uncropped version of all immunoblots found in supplementary figures.

Supplementary Table 1: Echocardiographic measurements after 14 days of treatment

		WT				AMPKα2 KO			
		controls	met	AngII	AngII+met	controls	met	AngII	AngII+met
**B mode**									
	**LV mass (mg)**	105.6±5.2	107.1±4.1	136.9±3.0*	111.1±4.2#	105.2±2.2	103.2±6.3	141.5±4.1*	136.4±5.7*
	**HW/TL (ratio)**	5.1±0.2	5.3±0.2	6.8±0.2*	5.5±0.3#	5.3±0.1	5.1±0.3	7.2±0.3*	7.1±0.3*
	**BW (g)**	27.5±0.8	28.8±2.4	28.0±0.7	26.1±0.6	27.6±0.7	29.6±1.7	28.0±0.9	27.5±0.7
	**HR (bpm)**	456±29	482±13	478±15	481±34	472±28	448±26	538±20	517±11
	**LVEDV (µl)**	65.0±1.3	69.7±5.4	58.9±3.2	59.9±3.2	58.0±3.2	60.6±2.6	62.0±1.6	56.2±4.0
	**LVESV (µl)**	32.5±1.7	33.6±3.6	28.2±1.7	30.9±1.4	28.2±1.4	29.9±2.0	33.8±1.3	33.3±2.9
	**LV EF (%)**	50.1±2.2	52.2±2.0	52.1±1.2	48.1±1.1	51.1±1.5	50.7±2.1	45.6±1.4	41.3±1.7*
	**FS (%)**	20.0±1.0	25.8±2.6	25.6±0.9*	22.1±1.3	21.1±1.4	26.8±2.2	20.9±1.2$	19.6±1.3$
**M mode**									
	**IVSd (mm)**	0.71±0.04	0.78±0.08	0.85±0.04	0.84±0.05	0.68±0.03	0.69±0.04	0.95±0.04*$	0.83±0.06
	**IVSs (mm)**	1.02±0.08	1.08±0.07	1.12±0.04	1.04±0.05	0.97±0.03	0.95±0.10	1.23±0.05*$	1.00±0.05#
	**LVIDd (mm)**	4.28±0.07	4.33±0.11	3.92±0.13	3.78±0.09*$	4.16±0.13	3.97±0.10	3.88±0.09	3.86±0.08
	**LVIDs (mm)**	3.43±0.08	3.21±0.12	2.92±0.12*	2.95±0.11	3.29±0.14	2.90±0.12	3.08±0.10	3.10±0.09
	**LVPWd (mm)**	0.67±0.05	0.83±0.10	0.86±0.06	0.95±0.08	0.74±0.05	0.78±0.05	0.97±0.08	0.87±0.04
	**LVPWs (mm)**	0.89±0.05	1.05±0.04	1.15±0.07	1.14±0.08	0.94±0.05	1.07±0.07	1.17±0.09	1.16±0.08

LV = left ventricle, HW/TL = heart weight/tibia length, BW = body weight, HR = heart rate, LVEDV = left ventricular end-diastolic volume, LVESV = left ventricular end-systolic volume, EF = ejection fraction, FS = fractional shortening, IVSd = end-diastolic interventricular septal thickness, IVSs = end-systolic interventricular septal thickness, LVIDd = end-diastolic left ventricular internal diameter, LVIDs = end-systolic left ventricular internal diameter, LVPWd = end-diastolic left ventricular posterior wall thickness, LVPWs = end-systolic left ventricular posterior wall thickness, AngII = angiotensin II, Met = metformin. The data are expressed as mean ± s.e.m.

*$p<0.05$ vs respective controls

#$p<0.05$ vs respective AngII condition

$\$p<0.05$ vs respective metformin condition

Supplementary Table 2: List of antibodies used in western blot, immunoprecipitation and immunofluorescence staining

Primary antibody	Company	Catalogue #	WB	IP	IF
p-AMPK (Thr172)	Cell Signaling	2531	1/1000		
AMPK alpha	Cell Signaling	2532	1/1000		
p-ACC (Ser79)	Cell Signaling	3661	1/1000		
ACC	Cell Signaling	3662	1/1000		
p-eEF2 (Thr56)	Cell Signaling	2331	1/2000		
eEF2	Cell Signaling	2332	1/1000		
p-ERK1/2 (Thr202/Tyr204)	Cell Signaling	9101	1/1000		
ERK1/2	Cell Signaling	9102	1/1000		
GFAT	Cell Signaling	5322 (D12F4)	1/2000		
OGT	Cell Signaling	5368	1/1000		
GAPDH	Cell Signaling	2118 (14C10)	1/50000		
O-GlcNAc RL2	Abcam	ab32739	1/1000		
O-GlcNAc RL2-HRP	Abcam	ab201995	1/1000		
p-GFAT (Ser243)	Dundee University	/	1/100		
OGA	Santa Cruz	sc-135093	1/1000		
TnT	Santa Cruz	sc-20025	1/2000	1/5000	
NFATc3	Santa Cruz	sc-8405			1/50
Alpha actinin	Sigma	A7811 (EA-53)			1/750

Secondary antibody	Company	Catalogue #	WB	IF
Anti-rabbit IgG-HRP	Sigma	A0545	1/20000	
Anti-mouse IgG-HRP	BD Biosciences	554002	1/5000	
Anti-sheep IgG-HRP	Sigma	A3415	1/20000	
Alexa Fluor 594 donkey anti-mouse	Invitrogen	A21203		1/1000
Alexa Fluor 488 donkey anti-mouse	Invitrogen	A21206		1/1000

WB, western blot; IP, immunoprecipitation; IF, immunofluorescence; HRP, horse radish peroxidase
